# Identification of Pristine and Protein Corona Coated Micro- and Nanoplastic Particles with a Colorimetric Sensor Array

**DOI:** 10.1021/acsomega.4c06166

**Published:** 2024-08-05

**Authors:** Shaun Grumelot, Ali Akbar Ashkarran, Zahra Jiwani, Rula Ibrahim, Morteza Mahmoudi

**Affiliations:** Department of Radiology and Precision Health Program, Michigan State University, East Lansing, Michigan 48824, United States

## Abstract

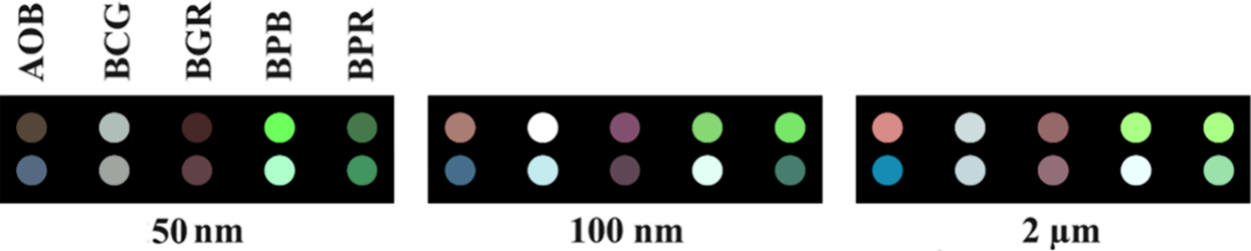

A colorimetric sensor array has been developed to differentiate various micro- and nanoplastic particles (MNPs), both pristine and those coated with a protein corona, in buffered water. This array utilizes five distinct cross-reactive chemo-responsive dyes, which exhibit changes in visible optical absorbance upon interaction with MNPs. Although no single dye responds exclusively to either pristine or protein-corona-coated MNPs, the collective shifts in color across all dyes create a unique molecular fingerprint for each type of MNP. This method demonstrates high sensitivity, capable of detecting MNPs of various sizes (50 nm, 100 nm, and 2 μm) and differentiating them from controls at concentrations as low as 10 ng/mL using standard chemometric techniques, ensuring accurate results without error. Additionally, the method can effectively distinguish between pristine and protein-corona-coated polystyrene MNPs. This colorimetric approach offers a rapid, cost-effective, and accurate method for monitoring MNP pollution and assessing their prior interactions with biological systems.

## Introduction

Plastic, a synthetic polymer, is widely utilized across various industries such as textiles, electronics, construction, packaging, and healthcare due to its versatility, affordability, moldability, durability, and strength.^1^ Annually, over 430 million tons of plastic are produced globally, and its production is consistently rising.^2^ The extensive use of plastic raises environmental concerns, particularly its decomposition. Plastics do not completely degrade, leaving behind persistent small fragments that contribute to pollution.^3^ The breakdown of plastic results in the formation of micro- and nanoplastics (MNPs).

The origins of MNPs are categorized into primary and secondary sources. Primary MNPs are industrial byproducts directly entering the ecosystem in their original size, as found in pharmaceutical items and personal care products.^4,5^ Secondary MNPs, such as those from food containers and tires, arise from the breakdown of larger plastic debris through physical stress, animal activities, microbial action, and other mechanisms.^5,6^ These MNPs, characterized by their small size and slow decomposition rate, can easily infiltrate and accumulate in water and air, posing a risk to organisms and ecosystems alike.^7^

As of 2016, it is estimated that between 19 and 23 million metric tons of plastic have entered aquatic environments.^8^ MNPs also originate from the global nanomaterials market, which includes a wide range of plastic-based NPs (e.g., polystyrene NPs), accounted for more than 1.6 million metric tons in 2020 and is projected to nearly double, reaching approximately 3.5 million metric tons by 2031.^9^ MNPs are a significant concern for soil contamination and can leach into groundwater, releasing harmful chemicals that affect both water resources and aquatic life.^10^ The widespread issue of improper plastic disposal exacerbates this problem, with approximately 80.5 million tons of plastic polluting the environment.^11^ Due to their small size, MNPs pose a global environmental threat, easily transitioning between aquatic and atmospheric systems. For instance, water contaminated with MNPs can evaporate and carry these particles into the atmosphere.

While our study acknowledges the extensive variety of MNPs available, our primary focus will be on MNPs originating from the global nanomaterials market.

Detecting low concentrations of MNPs in water resources presents a substantial challenge in environmental science. The isolation and quantification of nanoplastics in real-world samples are both difficult and crucial tasks. While techniques such as Fourier transform infrared spectroscopy and hyperspectral stimulated Raman scattering microscopy are available for identifying and measuring nanoplastics in environmental settings, there is an urgent need for simpler and faster methods to efficiently detect these pollutants.^12,13^ Additionally, understanding the “biological memory” of MNPs—whether they have interacted with a biosystem such as biological fluids—is critical for tracing MNPs’ potential pathways and pinpointing their sources of release into the environment.^14^

Optical sensors, particularly colorimetric sensor arrays, have emerged as rapid, sensitive, and adaptable tools for analyzing liquids, vapors, and gases.^15,16^ Their effectiveness lies in the collective pattern of responses from cross-reactive sensor arrays, not from individual sensors targeting specific analytes.^16^ These arrays have differentiated among a wide variety of analytes, including toxic industrial chemicals, explosives, various foods and beverages, pathogenic microorganisms, and even nanoparticles.^17,18^

In this study, we employ a colorimetric sensor array that utilizes five water-soluble chemoresponsive dyes—bromocresol green (BCG), bromophenol blue (BPB), bromophenol red (BPR), bromopyrogallol red (BGR), and acridine orange base (APB)—for the detection and identification of polystyrene MNPs of various sizes: 20 nm, 100 nm, and 2 μm. Additionally, we investigated the capability of the colorimetric sensor array to determine whether the system can detect and discriminate between MNPs with and without interactions with biological fluids.

## Materials and Methods

### Materials

Phosphate-buffered saline (PBS, 10×) pH 7.4 and fetal Bovin serum (FBS) were purchased from Thermo Fisher Scientific. Various dyes used for developing the colorimetric sensor array, including bromopyrogallol red (5 g), bromocresol green (0.04% aqueous solution), bromophenol blue (0.04% aqueous solution), bromophenol red (5 g), and acridine orange (10 mg/mL ≥ 95% in water), were acquired from Avantor. Micro- and nanoplastic particles (MNPs) were sourced from Polysciences.

### Protein Corona Formation on the Surface of MNPs

For protein corona formation, FBS 55% were mixed with MNPs (final concentration of 0.2 mg/mL) and incubated for 1 h at 37 °C. To remove unbound and FBS proteins only loosely attached to the surface of MNPs, protein–MNPs complexes were then centrifuged at 12,000×*g* for 30 min, the collected MNPs’ pellets were washed two more times with cold PBS under the same conditions, and the final pellet was collected for applications in sensor array.

### Characterization

Optical absorption spectra of the MNPs solutions were measured using a Spectra max M2 UV–vis-NIR spectrophotometer. Utilizing the UV–vis absorption spectrum, initial estimates of the MNPs’ size, concentration, and biological interactions were determined. Additionally, sodium dodecyl sulfate–polyacrylamide gel electrophoresis (SDS-PAGE) was conducted on the protein-corona-coated MNPs to visualize the patterns of the protein corona. For each SDS-PAGE experiment, 20 μL of bare MNPs (as control) and protein corona-coated MNPs at various concentrations were combined with 20 μL of 2× Laemmli sample buffer, heated at 85 °C for 6 min, and loaded into the precast gels. Following gel electrophoresis, the gels were fixed in a solution containing 10% acetic acid and 40% ethanol, then stained overnight with 50 mL of Coomassie blue stain. After multiple washes, the gels were scanned the next morning. Transmission electron microscopy (TEM) analysis was performed using a JEM-2200FS (JEOL Ltd.) operated at 200 kV. The instrument was equipped with an in-column energy filter and an Oxford X-ray energy dispersive spectroscopy (EDS) system to enhance imaging and analytical capabilities. For imaging, approximately 5 μL of the bare MNPs solution was deposited onto a copper grid and analyzed under electron microscope.

## Results and Discussion

**Figure 1:**
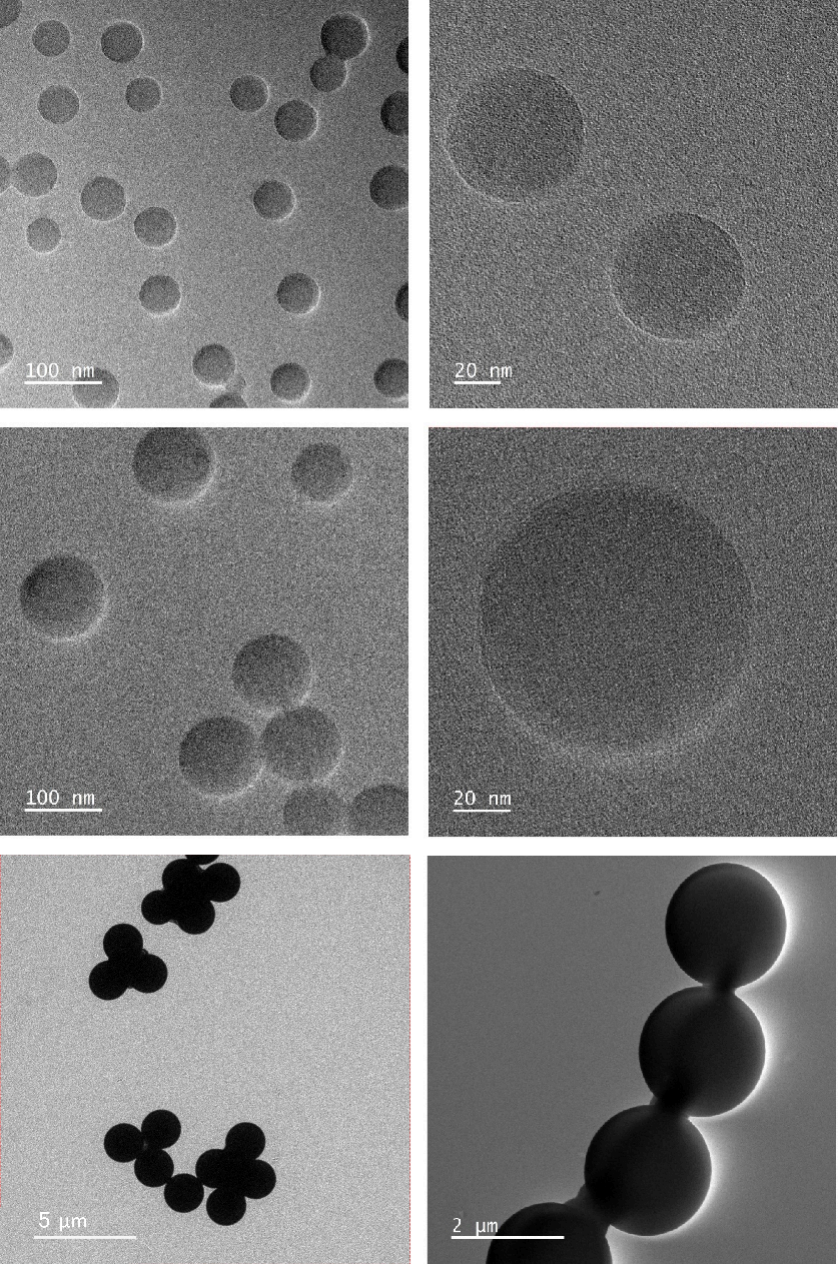
Representative TEM images showcasing MNPs at various scales. Top panels display 50 nm MNPs, the middle panels show 100 nm MNPs, and the bottom panels illustrate 2 μm MNPs.

We focused on a specific group of MNPs—narrow sized polystyrene particles of sizes 50 nm, 100 nm, and 2 μm (Figure 1)—along with necessary controls, chosen for their widespread synthesis and application across numerous fields. Traditionally, the colorimetric sensor array method involves using printed dye arrays on porous membranes.^16^ However, for detecting MNPs, we utilized an array of solution-phase sensors and created color difference maps by tracking the visible optical absorbance spectrum changes in the dyes when exposed to MNPs in aqueous solutions. We successfully used this approach for the quick and precise detection of gold NPs with various physicochemical properties in water.^18^

To guarantee accurate assessment of the dyes’ color shifts post-MNP interaction, we meticulously controlled the pH at 7.41 using standard phosphate-buffered saline (PBS) solutions. We prepared dye solutions at varying concentrations and exposed them to different MNP concentrations (ranging from 10 to 1000 ng/mL) at a pH of 7.41. The visible light spectra of these solutions were then meticulously collected and analyzed.

**Figure 2:**
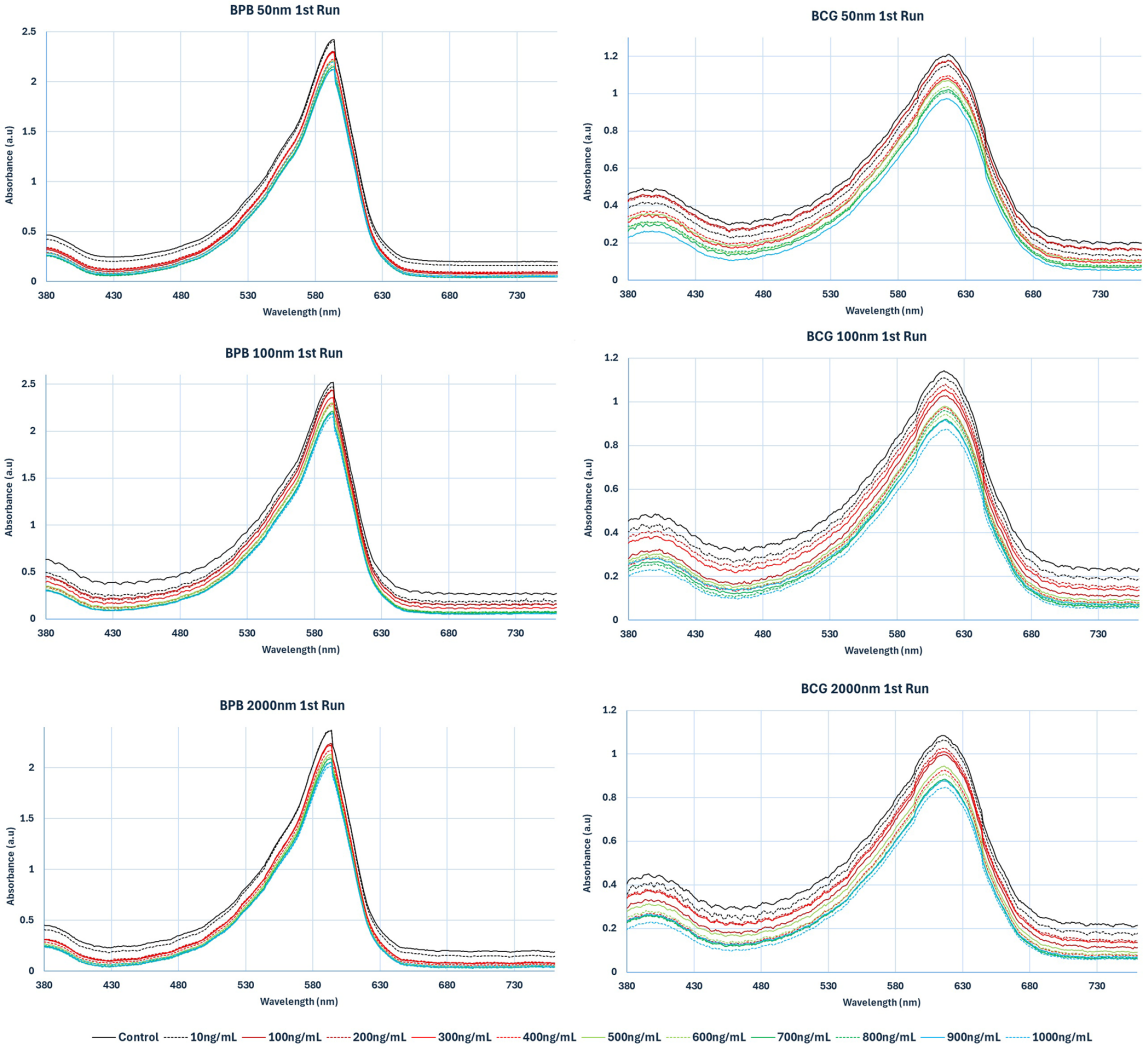
Representative absorbance spectrum of dyes as a function of MNP concentrations.

**Figure 3:**
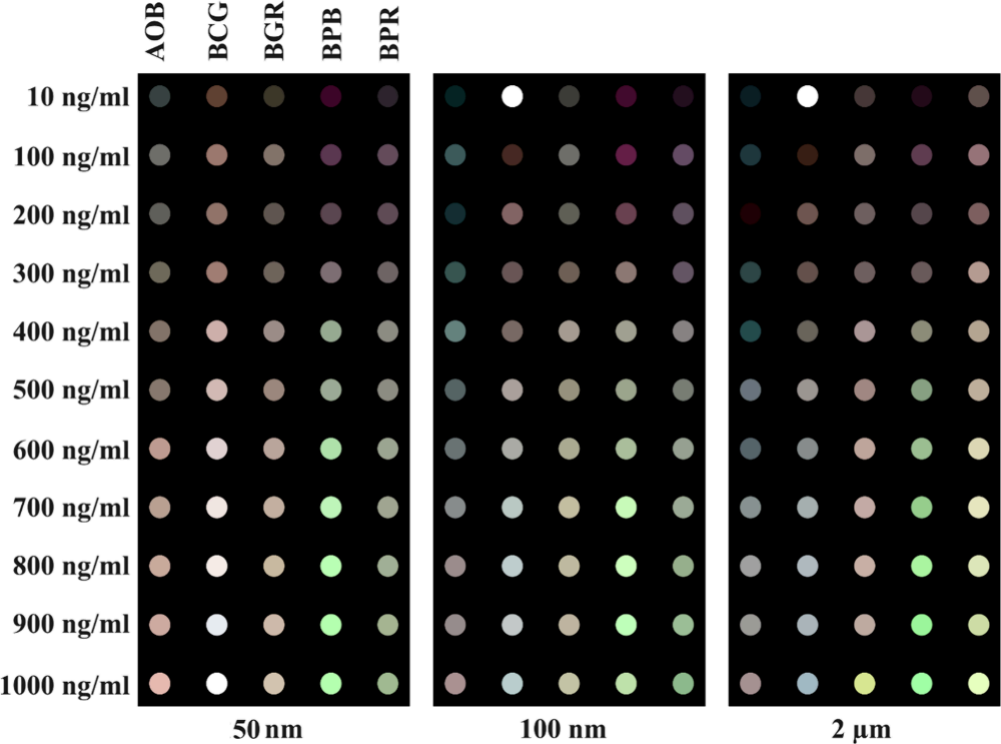
Color-change profiles of five sensor dyes following their interaction with various MNPs at different concentrations. For visualization, these color-difference maps were constructed by subtracting the absorbance of the solution prior to exposure from that after exposure to the MNPs at three chosen wavelengths (480, 590, and 620 nm), which correspond to RGB values. Absorbance at each of these wavelengths, ranging from 0 to 0.484 optical density, was linearly mapped to a scale of 0 to 255 in RGB values. Method effectively visualizes the intensity and pattern of color changes induced by MNPs at these specific wavelengths.

Figure 2 displays representative UV/vis patterns at specific concentrations for each MNP type. Color-difference maps were created for the dyes by subtracting the light absorbance values before exposure from those after exposure to MNPs at three selected wavelengths: 480, 590, and 620 nm. These wavelengths were chosen as they are near optimal for eliciting the maximum color changes in the dye spectra. As illustrated in Figure 3, the difference maps provide unique fingerprint patterns corresponding to the various dyes’ reactions to the NPs. These patterns effectively distinguish both the types of MNPs and their concentrations. Visually, even before any statistical analysis, each NP type can be identified by a distinctive pattern in the array response.

We maintained strict control over the pH of the solutions, ensuring a constant pH of 7.4 throughout our experiments. This stringent control confirms that the observed color changes in the sensor dyes are not attributable to fluctuations in the bulk pH of the solutions. Instead, these color variations must be attributed to changes in the local environment surrounding the dyes at the interface of the MNPs.

The interaction at the MNP-dye interface likely involves a variety of molecular interactions including local pH effects, Lewis acid–base interactions, hydrogen bonding, and changes in local polarity, known as solvatochromic effects.^16,19,20^ These interactions highlight the sensitivity of the dyes to the immediate microenvironment, providing a vivid illustration of the physicochemical dynamics at the MNP interface. The nature and behavior of this interfacial region are reflective of the specific physicochemical properties inherent to each type of MNP. Consequently, analyzing these interfacial interactions not only helps in identifying the MNPs but also in understanding the underlying mechanisms driving these interactions. Such insights are crucial for developing more sophisticated and targeted approaches for MNP detection and characterization in various environmental and biological contexts.

**Figure 4:**
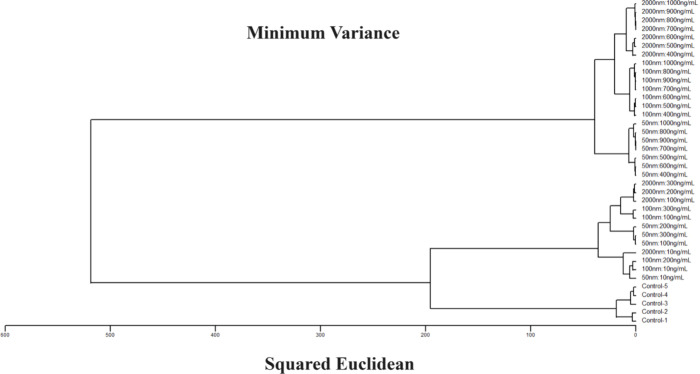
HCA dendrogram illustrating the color changes in sensor dyes upon exposure to various MNPs at different concentrations, compared to controls without MNPs. Analyte labels specify MNP identity and concentration, with all experiments conducted in triplicate. No classification errors were observed among MNPs at concentrations of 400 ng/mL. At concentrations of 200 ng/mL and below, minor misclustering occurred among different sizes of MNPs; however, even at the lowest tested concentration of 10 ng/mL, the MNPs were still distinguishable from the control samples. Clustering was performed using the minimum variance method (Ward’s method^22^).

To further evaluate the capability of our colorimetric sensor array to distinguish between different types of MNPs, we employed hierarchical cluster analysis (HCA). Unlike model-dependent techniques such as linear discriminant analysis or support vector machine analysis, HCA is a model-free statistical method that operates without making any a priori assumptions about the class identities of the data.^21^ The dendrogram resulting from this analysis is presented in Figure 4, demonstrating complete and accurate discrimination among all the tested MNP types.

**Figure 5:**
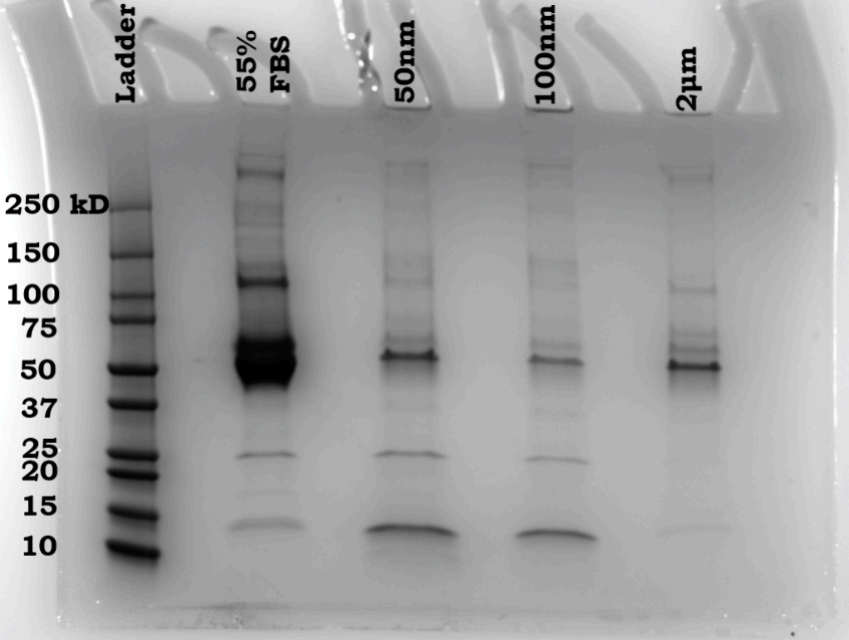
Representative SDS-PAGE image of the protein corona profiles of MNPs.

**Figure 6:**
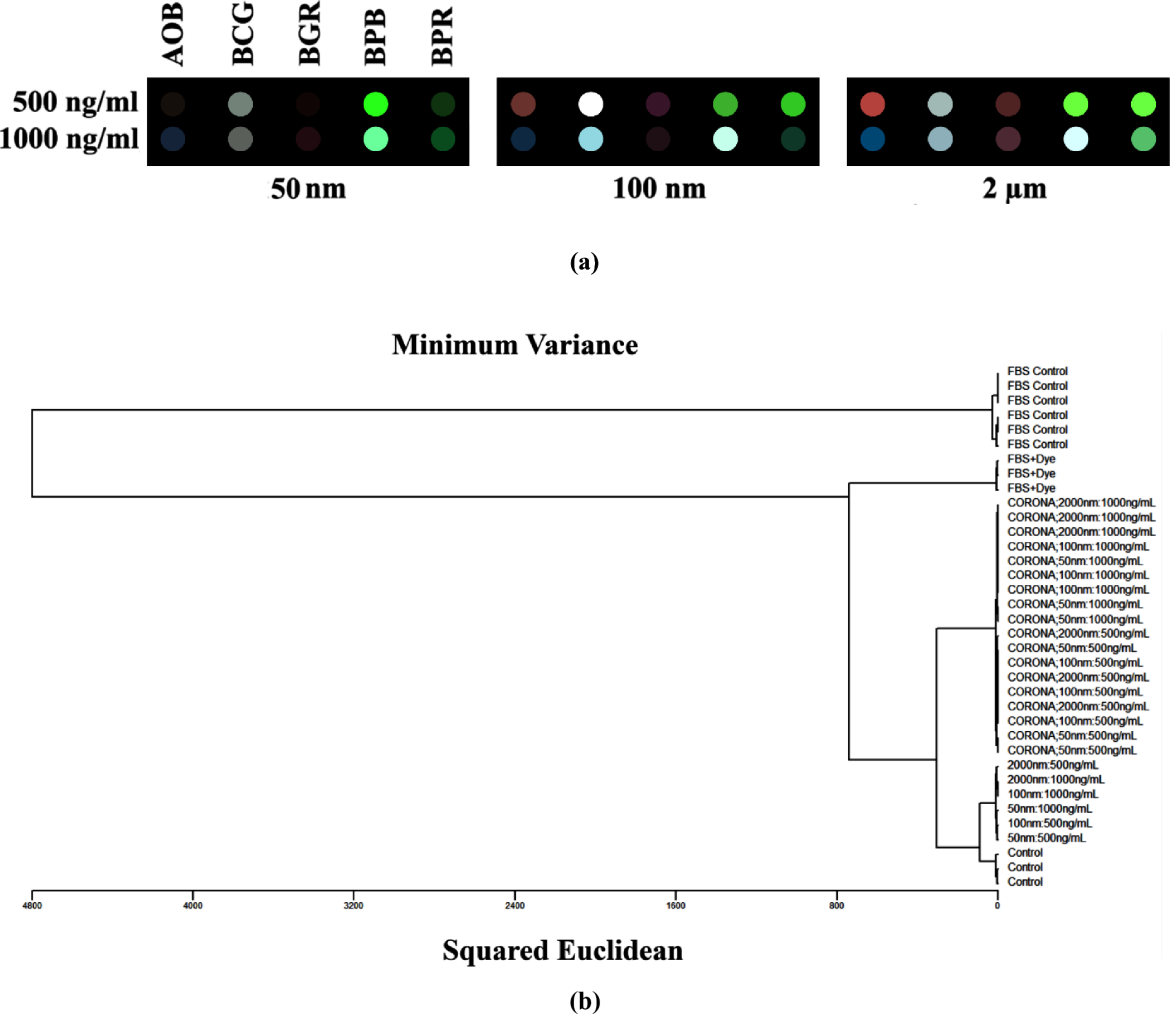
(a) Color-change profiles of five sensor dyes after interacting with various corona-coated MNPs. These profiles display the sensor responses to different types of MNPs. (b) HCA dendrogram depicting the color responses of sensor dyes to MNPs at concentrations of 500 and 1000 ng/mL, both with and without protein corona coatings (the label “-CORONA” denotes protein-corona-coated MNPs), compared to control samples (i.e., protein source) without MNPs. Analyte labels indicate MNP identity and concentration, with all experiments performed in triplicate. No misclassifications were observed among pristine, protein-corona-coated MNPs, and controls. Clustering was conducted using the minimum variance method (Ward’s method).

Next, we explored whether our sensor array could detect MNPs that had acquired biological memories. To this end, we prepared protein-corona-coated MNPs by incubating them with 55% fetal bovine serum (FBS); Figure 5. We then subjected both the resultant hard corona-coated MNPs and essential controls—including uncoated MNPs, FBS alone, and FBS mixed with dyes—to analysis using our sensor array at two concentrations (500 and 1000 ng/mL), as representatives. Color-difference maps were generated to illustrate the interactions between these protein corona-coated MNPs and the dyes. As depicted in Figure 6a, these maps displayed unique fingerprint patterns that corresponded to the dyes’ varied responses to the protein corona-coated MNPs. Similar to their pristine counterparts, each MNP type was visually identifiable by a distinctive pattern in the array response, even prior to any statistical analysis.

Further analysis was performed using HCA on the protein-corona-coated MNPs and controls. The dendrogram resulting from this analysis, shown in Figure 6b, demonstrated complete and accurate discrimination among all the tested protein-corona-coated MNP types and controls, with no instances of misclassification.

## Conclusions

In summary, this study has successfully demonstrated the effectiveness of a colorimetric sensor array for detecting and differentiating various types and concentrations of micro- and nanoplastic particles (MNPs), both pristine and protein-corona-coated, in buffered water environments. Utilizing five water-soluble chemoresponsive dyes, the sensor array provided detailed color-change profiles that serve as unique molecular fingerprints for each type of MNP. These fingerprints allow for the rapid and accurate identification of MNPs, highlighting the array’s high sensitivity and potential for practical application in environmental monitoring. The findings highlights the importance of advanced sensor technologies in addressing environmental challenges, particularly the pervasive issue of plastic pollution. By offering a method that simplifies the detection of MNPs and sheds light on their interactions with biological systems, this research contributes valuable insights into the pathways and impacts of MNPs in aquatic ecosystems. The ability to trace MNPs that have acquired biological memories further enhances our understanding of their environmental behavior and potential health implications. Future research should focus on expanding the types of MNPs that can be detected by the sensor array, as well as exploring the array’s application in other environmental matrices such as soil and air. Additionally, further refinement of the chemometric techniques used could improve the discrimination accuracy at lower concentrations and for MNPs with similar physicochemical properties. Ultimately, the development and application of such innovative technologies are crucial for advancing our ability to monitor and mitigate the environmental risks associated with nanoscale pollutants. This study not only contributes to the field of environmental science but also paves the way for future innovations in the detection and management of environmental pollutants.
